# Genome-wide polygenic risk score for major osteoporotic fractures in postmenopausal women using associated single nucleotide polymorphisms

**DOI:** 10.1186/s12967-023-03974-2

**Published:** 2023-02-16

**Authors:** Qing Wu, Jongyun Jung

**Affiliations:** Department of Biomedical Informatics, College of Medicine, The Ohio State University, 250 Lincoln Tower, 1800 Cannon Drive, Columbus, OH 43210 USA

**Keywords:** GPS, GRS, GWAS, Fracture, Osteoporosis, Bone density

## Abstract

**Background:**

Osteoporosis is highly polygenic and heritable, with heritability ranging from 50 to 80%; most inherited susceptibility is associated with the cumulative effect of many common genetic variants. However, existing genetic risk scores (GRS) only provide a few percent predictive power for osteoporotic fracture.

**Methods:**

We derived and validated a novel genome-wide polygenic score (GPS) comprised of 103,155 common genetic variants to quantify this susceptibility and tested this GPS prediction ability in an independent dataset (n = 15,776).

**Results:**

Among postmenopausal women, we found a fivefold gradient in the risk of major osteoporotic fracture (MOF) (p < 0.001) and a 15.25-fold increased risk of severe osteoporosis (p < 0.001) across the GPS deciles. Compared with the remainder of the GPS distribution, the top GPS decile was associated with a 3.59-, 2.48-, 1.92-, and 1.58-fold increased risk of any fracture, MOF, hip fracture, and spine fracture, respectively. The top GPS decile also identified nearly twofold more high-risk osteoporotic patients than the top decile of conventional GRS based on 1103 conditionally independent genome-wide significant SNPs. Although the relative risk of severe osteoporosis for postmenopausal women at around 50 is relatively similar, the cumulative incident at 20-year follow-up is significantly different between the top GPS decile (13.7%) and the bottom decile (< 1%). In the subgroup analysis, the GPS transferability in non-Hispanic White is better than in other racial/ethnic groups.

**Conclusions:**

This new method to quantify inherited susceptibility to osteoporosis and osteoporotic fracture affords new opportunities for clinical prevention and risk assessment.

**Supplementary Information:**

The online version contains supplementary material available at 10.1186/s12967-023-03974-2.

## Background

Osteoporosis is a common bone disease characterized by decreased bone mass and deterioration of bone microstructure, leading to decreased bone strength and increased risk of fragility fracture [1]. Fragility fracture has become a rapidly growing public health issue affecting more than 8.9 million people worldwide [2], as osteoporosis is a metabolic disease uniquely associated with aging. With life expectancy increasing worldwide, osteoporosis, fragility fractures, and the subsequent devastating consequences of fractures continue to be a growing global burden of morbidity, mortality, and socioeconomic cost.

Osteoporosis is highly heritable, with heritability ranging from 50 to 85% [3]. Most inherited susceptibility is associated with common DNA variants [3]. Genome-wide association studies (GWASs) and GWAS meta-analyses have discovered hundreds of loci, including thousands of Single Nucleotide Polymorphisms (SNPs), associated with osteoporosis, bone mineral density (BMD) and osteoporotic fractures [4–6]. Although these discovered SNPs are significantly and robustly associated with osteoporotic fracture and related traits, previous efforts to create an effective genetic risk score (GRS) for osteoporotic fracture and BMD have had only modest success [7–9]. These reported GRS only explained a small percentage variance of BMD and osteoporotic fracture, thus providing limited predictive power for fracture outcomes [8]. Studies found that the clinical utility of these reported GRS in fracture prediction and risk assessment is substantially low [8–10].

Therefore, we aimed to utilize the recently developed computational algorithms [11] and incorporate a large GWAS summary dataset [4] to derive, validate, and test a new genome-wide polygenic score (GPS) to improve fracture prediction. This new GPS integrates all available common variants into a single quantitative measure of inherited susceptibility. The newly developed GPS can simultaneously identify a subset of postmenopausal women at substantially higher risk of severe fracture, as well as lower fracture risk. We hypothesize that this novel GPS could provide much higher prediction power for osteoporotic fracture and, therefore, could be utilized to improve fragility fracture prediction.

## Methods

### Study cohorts

The Women Health Initiative (WHI) study is the US nationwide, long-term health study in postmenopausal women, with fragility fracture as one of the major outcomes. From 1993 to 1998, the WHI enrolled 161,808 women aged 50 to 79 in randomized clinical trials (CT) or an observational study (OS). The WHI OS examined predictors and important causes of morbidity and mortality [12], while WHI CT examined the effects of menopausal hormone therapy (HT) vs. placebo, calcium and vitamin D supplementation vs. placebo, and low-fat eating patterns vs. usual eating patterns. Participants were provided by mail or telephone with questionnaires annually in the observational study or semiannually in the clinical trials.

This study utilized data from the four WHI sub-studies: Genomics and Randomized Trials Network (GARNET) (https://www.ncbi.nlm.nih.gov/projects/gap/cgi-bin/study.cgi?study_id=phs000315.v8.p3), Integrative genomics and risk of coronary heart disease and related phenotypes (https://www.ncbi.nlm.nih.gov/projects/gap/cgi-bin/study.cgi?study_id=phs001335.v2.p3), Population Architecture using Genomics and Epidemiology (PAGE) (https://www.ncbi.nlm.nih.gov/projects/gap/cgi-bin/study.cgi?study_id=phs000227.v5.p3), and Women’s Health Initiative Memory Study (WHIMS) (https://www.ncbi.nlm.nih.gov/projects/gap/cgi-bin/study.cgi?study_id=phs000675.v4.p3). DNA samples were processed from whole blood collected at a dedicated research center. Samples have been genotyped using the Illumina (Illumina Inc., San Diego, CA, USA) or Affymetrix 6.0 Array Set Platform (Affymetrix Inc., Santa Clara, CA, USA).

Whole blood samples at the baseline were used for DNA extraction. Consent for DNA use was obtained through written permission. We used minor allele frequency $\ge 0.01$, individual missing value rate $<5\%$, SNPs call rate $>95\%$, and Hardy–Weinberg equilibrium $p$ value $<0.0001$ as a quality-control criterion. The quality control of genotype data was performed using Plink [13]. When multiple probes measured the identical genotypes, multiple probes were checked for concordance and were set to a missing value if the genotypes did not match. Then files were converted to variant call format (VCF), separated by chromosomes. Genetic imputation was conducted using the TOPMed reference panel [14] and the Michigan Imputation Server [15]. For the present analysis, up to 19,515 participants with major osteoporotic fracture and genotyping array data were available.

#### Informed consent and study approval

The WHI’s participants were recruited from areas surrounding forty clinical centers established primarily at major academic health centers in 24 states and the District of Columbia [16]. The Institutional Review Board of each participating institution approved study protocols and consent forms [17]. At the beginning of the first screening visit, each woman was given general information about the WHI components and viewed an introductory video providing an overview of the study. An informed consent form was signed to cover initial screening activities, including processing questionnaire data, drawing blood, and obtaining medical records.

The datasets used in this analysis were accessed with appropriate approval through the database of Genotype and Phenotype (dbGap) online resource [18] with accession number phs000200.v12.p3 and the approval of the institutional review board at the Ohio State University.

## Method details

### Polygenic score derivation and validation

For the new score derivation, we used published summary statistics from a recent genome-wide association study (GWAS) for bone mineral density estimated from quantitative heel ultrasounds (eBMD) [4], including genotyping and imputed data in up to 426,824 participants of the UK Biobank study available for download from the GEnetic Factor for OSteoporosis consortium (GEFOS) website [19]. The summary statistics of genetic association were available for 13,753,401 SNPs for eBMD. To minimize the computational burden and have a high-quality variant, we performed the quality control of summary statistics only, including the imputation quality score (INFO) > 0.8 and $p\le 5\times {10}^{-8}$, which resulted in 103,155 variants. The linkage disequilibrium reference panel of 503 European samples from 1000 Genomes phase 3 version 5 [20] was employed to incorporate the correlated variants. DNA polymorphisms with an ambiguous strand (A/T or C/G) were removed from the score derivation.

Seven candidate polygenic scores were derived using the LDPred computation algorithm [11]. This Bayesian approach calculates a posterior mean effect size for each variant based on a prior and subsequent shrinkage based on the extent to which this variant is correlated with similarly associated variants in the reference population. The underlying Gaussian distribution additionally considers the fraction of causal (i.e., non-zero effect sizes) markers via a tuning parameter, $\rho$. Because $\rho$ is unknown for any given disease, a range of $\rho$ values, the fraction of causal variants, was used 0.001, 0.003, 0.01, 0.03, 0.1, 0.3, and 1.

An eighth score was derived with 1,103 conditionally independent variants identified from the previously published GWAS study of summary statistics results [4]. We computed the eighth score using the linkage disequilibrium-based clumping procedure in PLINK version 2.0. The algorithm identifies a list of independent $({r}^{2}<0.2)$ variants and 1103 associated SNPs were extracted and analyzed. The conventional genome-wide significant variants score was calculated as a weighted sum of risk alleles ${\sum }_{i}\widehat{{\beta }_{i}}{x}_{i}$, where ${x}_{i}$ is the expected number of risk alleles and $\widehat{{\beta }_{i}}$ is the log-odds ratio estimate of single-variant association from the GWAS result [4].

The eight scores were calculated in a validation dataset of 2,458 participants of the GARNET WHI sub-study. More than 99% of variants in the GPSs were available for scoring purposes in the validation dataset with an excellent imputation quality score (INFO > 0.8). The polygenic score with the strongest correlation with observed BMD (hip and spine) in the validation dataset was determined based on Pearson correlation, and the best score was carried forward into subsequent analyses in an independent testing dataset of 15,776 participants.

Since the independent testing dataset (n = 15,776) contains diverse ancestry participants, which includes American Indian or Alaskan Native, 2.5%; Asian or Pacific Islander, < 1%; Black or African-American, 57%; Hispanic/Latino, 18.6%; Not-Hispanic White, 21.7%), we take advantage of using ancestry-specific polygenic score approach by using the principal component analysis (PCA) since the ancestry-specific polygenic score approach provides a better estimation when different ancestry participants are included in the data set [21].

To estimate genetic ancestry, we performed the PCA with EIGENSTRAT software to obtain principal components (PCs) to measure genetic ancestry [22, 23]. The EIGENSTRAT method uses PCA to explicitly model ancestry differences across ancestral populations, minimizing spurious associations while maximizing the power to detect genuine associations [22]. We obtained the top ten PCs for each participant in our independent testing dataset. We used the 103,155 genetic variants to calculate the unadjusted seven candidate GPS with a different fraction of causal variants (Eq. 1). An adjusted seven-candidate GPS was calculated with the 103,155 genetic variants, adjusting for the top ten PCs of genetic ancestry to predict the ancestry-specific polygenic score (Eq. 2).

1 $${\text{GPS}}_{\text{unadj}}= \sum_{i=1}^{\mathrm{103,155}}\widehat{{\beta }_{i}}{\text{SNP}}_{i}$$

2 $${\text{GPS}}_{\text{adj}}= \sum_{i=1}^{\mathrm{103,155}}\widehat{{\beta }_{i}}{\text{SNP}}_{i}+ \sum_{i=1}^{10}{\text{PC}}_{i}$$

We calculated the residual between Eqs. 1 and 2. We adjusted the residual in each GPS to create an ancestry-corrected polygenic score. Throughout our study, we used an ancestry-corrected polygenic score in all primary analyses.

### WHI Phenotypes

At present, the osteoporosis diagnostic criteria were established by the World Health Organization (WHO) [24]. Osteoporosis is diagnosed by central dual-energy x-ray absorptiometry (DXA) measurement if the T-score of the spine is -2.5 or less [25, 26]. Within the WHI cohorts, using the observed spine BMD and reference value based on Looker et al.’s study [27], we calculated T-score for each participant. We defined a participant as having normal BMD if a participant’s T-score $\ge -1$, osteopenia if a participant’s T-score is between − 1 and − 2.5, osteoporosis if a participant’s T-score $\le -2.5$, and severe osteoporosis if a participant present with one or more fragility fracture (s) and a T-score $\le -2.5$.

A major osteoporotic fracture (MOF) was defined as a fracture of the hip, spine (clinical), forearm, or shoulder. The WHI participants were followed for 12 years on average from the baseline examination. The follow-up period was calculated from the enrollment or randomization to the time of the first fracture or death. People who did not experience a fracture or death were followed until the end of the initial WHI study. Self-reported fracture outcomes were identified by questionnaires semiannually for CT participants and annually for OS participants.

Age and race/ethnicity were collected using the pre-designed questionnaire at the baseline. Participants in WHI enrolled at three clinical centers (Pittsburgh, PA; Birmingham, AL; and Tucson/Phoenix, AZ, USA) and performed dual-energy x-ray absorptiometry (DXA) measurement of the lumbar spine and hip BMD using a Hologic machine (QDR 2000, 2000 + , or 4500W, Hologic Inc, Bedford, Mass). Women were excluded at these three BMD centers if their femoral neck BMD was more than three standard deviations below the corresponding age-specific mean (Z score $\le -3.0$) [28]. The baseline BMD measurement was used for this study. Participants’ weight and height were measured in the clinic using standardized protocols. Parental fracture is determined by if a participant’s father or mother had a fracture. The previous fracture is ascertained if a participant had an osteoporosis-related fracture or broken bone. Glucocorticoid use is defined as if a participant had taken a glucocorticosteroid orally and daily. Rheumatoid arthritis is defined if a participant who has had rheumatoid arthritis ever. Previous osteoporosis is defined if a participant had osteoporosis ever before. Smoking was categorized into three groups; never smoked, past smoker, and current smoker.

### Quantification and statistical analysis

Genotyping array data was imputed (described above) within the three testing cohorts, and GPS was calculated for each individual. Genome-wide significant variants score was generated by multiplying the genotype dosage of each risk allele by its respective weight, then summing across all variants in the score. Incorporating genotype dosages accounts for uncertainty in genotype imputation. Scoring was completed using the PLINK2 software program with the –score command [29].

Within an independent testing dataset, participants were stratified according to the ten deciles of the GPS. Average BMD (hip and spine), weight, and prevalence of severe osteoporosis were determined within each decile. The association between high polygenic scores, defined as top deciles of the GPS, with severe osteoporosis was examined using multiple logistic regression in an independent testing dataset.

A multiple logistic regression model was employed to calculate the corresponding effect size (odds ratio). Our first multiple logistic regression model employed the clinical risk factors, adjusting for age, height, weight, parental fracture, previous fracture, smoking, glucocorticoid use, rheumatoid arthritis, hip BMD, and previous osteoporosis, along with GPS. In the second model, we only replaced spine BMD with hip BMD. Another same multiple logistic regression model was employed by only replacing GPS with GRS in the model.

In addition, we categorized an independent testing dataset into four different groups; top 30% vs. remaining 70%, top 20% vs. remaining 80%, top 10% vs. remaining 90%, and top 5% vs. remaining 95%. We estimated the odds ratio and 95% CI for individuals in the top 30%, 20%, 10%, and 5% of the GPS and GRS compared with the remaining individuals. We assessed the transferability of GPS by comparing the odds ratio and 95% CI estimates between the Non-Hispanic White, Black or African American, Hispanic/Latino, and others (including American Indian or Alaskan Native, and Asian or Pacific Islander). We estimated the odds ratio and 95% CI for individuals in the top 30%, 20%, 10%, and 5% of the GPS in the Non-Hispanic White, Black or African American, Hispanic/Latino, and others (including American Indian or Alaskan Native, and Asian or Pacific Islander), compared with the remaining individuals. To gauge the potential clinical impact of GPS, we calculated the prevalence of severe osteoporosis subjects by 20 years of follow-up in an independent testing dataset. GPS was stratified into top deciles, deciles 2–9, and bottom deciles.

To assess and compare the discriminative capacity of the GPS or GRS with clinical risk factors, we obtained Harrell C statistic [30] in an independent testing dataset (n = 15,776) using a multiple logistic regression model. The C statistics of individual clinical risk factors, GRS, or GPS were assessed on top of a baseline model of age, height, and weight in an independent testing dataset (n = 15,776).

Statistical analyses were conducted using R version 3.6.1 software (The R Foundation) [31]. A $p$-value of < 0.05 was considered statistically significant.

## Results

### GPS derivation and selection

To calculate a GPS, we used a recently developed computational algorithm, LDPred [11], to reweight each variant and obtain the average effects for each of 103,155 genetic variants on fracture and estimated BMD from the most extensive GWAS study of osteoporosis published to date [4]. This algorithm can reweight each variant according to the given effect size of the prior distribution and incorporate more variants observed in the prior GWAS. Moreover, with the input of a comprehensive reference panel, this method can incorporate the degree of correlation between a variant and others nearby and a tuning parameter that denotes the proportion of variants with non-zero effect size [11]. Vilhjálmsson et al. [11] recommended testing a range of different tuning parameters to capture the non-zero effect size. In our study, we tested the seven candidate tuning parameter values in order to identify the best GPS.

We tested the seven candidate-GPSs with measured hip and spine BMD in a validation dataset of 3739 women from the Women’s Health Initiative (WHI) Genomics and Randomized Trials Network (GARNET) study. The WHI GARNET study aimed to identify genetic factors affecting myocardial infarction, stroke, venous thrombosis, and diabetes phenotypes through genome-wide analysis using a nested case-control study design [32]. Of the 3739 women in the GARNET study, 2458 participants had genotype and BMD measurements available. All 2458 participants were Non-Hispanic White population. We compared the maximal correlation coefficient with each BMD to select the best GPS. Each candidate-GPS was strongly correlated with measured BMD at both spine and hip (all p < 0.001). The correlation coefficients ranged from − 0.22 to 0.21 for hip BMD (Additional file 1: Figure S1) and from 0.22 to 0.21 for spine BMD (Additional file 1: Figure S2), respectively. Similar results were obtained for each GPS after the adjustment of genetic background, which was evaluated and quantified by the top ten principal components of ancestry.

**Fig. 1:**
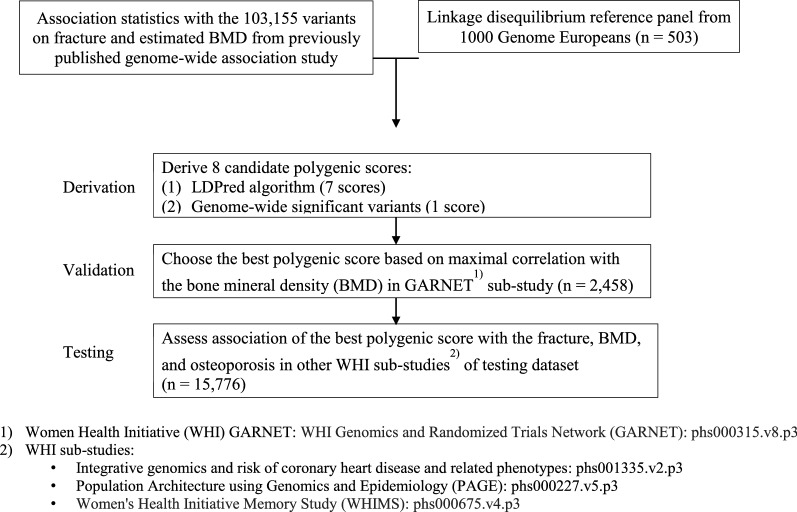
Study Overview. Derivation, Validation, and Testing of a Genome-wide Polygenic Score (GPS) for Osteoporosis. A genome-wide polygenic score for osteoporosis was used, including 103,155 variants from the previously published [4] GWAS study. The LDPred computational algorithm [11] was used to calculate GPS vary for the tuning parameter, $\rho$, the fraction of causal variants, 0.001, 0.003, 0.01, 0.03, 0.1, 0.3, and 1. A conventional genome-wide significant variants score with conditionally independent 1,103 variants from the previously published GWAS study [4]

The highest correlation with BMD in both spine (− 0.22) and hip (− 0.22) was achieved with the GPS ($\rho =0.03$); hence we used the GPS ($\rho =0.03$) as the best one to move forward in the analysis of the testing dataset. The best-performing GPS (ρ = 0.03) contains all 103,155 variants. The GPS (ρ = 0.03) is normally distributed with the empirical risk of fracture (Fig. 3). The median GPS (ρ = 0.03) percentile score was $-25$ for individuals without the fracture vs. 42 for individuals with the fracture. The testing dataset (n = 15,776) is independent of the validation dataset (n = 2458) studied above. Additional details of GPS derivation and validation are shown in Fig. 1.

Our GPS of 103,155 variants showed significantly higher predictive power than the conventional GRS, which comprises the conditionally independent 1103 SNPs [4] from a comprehensive GWAS study. Within the validation dataset ($$n=2458)$$, correlation with each BMD for GRS derived from 1103 SNPs [4] ranged from − 0.146 to − 0.159. This lower strength of association with BMD using only the conditionally independent, genome-wide significant 1103 SNPs was consistent with the previous study [33], ranging from − 0.154 to − 0.188. Having derived and validated a new polygenic score that considerably outperformed the conventional GRS calculated from the 1103 SNPs, we further studied the predictive power of the new GPS on major osteoporotic fracture, weight, and osteoporosis in an independent testing dataset (n = 15,776).

### Polygenic susceptibility to major osteoporotic fracture (MOF) and osteoporosis

We examined the extent to which the GPS predicted MOF and osteoporosis in an independent testing dataset (total n = 15,776) of WHI three sub-studies, including Integrative genomics and risk of coronary heart disease and related phenotypes, Population Architecture using Genomics and Epidemiology (PAGE), and Women’s Health Initiative Memory Study (WHIMS). This testing dataset is independent of the validation dataset (n = 2458) used earlier in this study. We also investigated the transferability of GPS in the independent testing dataset of 15,776 participants stratified by race/ethnicity: the Non-Hispanic White (n = 3427), Black or African-American (n = 9742), Hispanic/Latino (n = 2929) and others (n = 429, including American Indian or Alaskan Native, and Asian or Pacific Islander).

The baseline characteristics of the participants in an independent testing dataset were stratified by MOF status (Table 1). About 6% (n = 941) of participants had at least one major osteoporotic fracture (MOF) during an average of 12 years of follow-up. The participants with MOF have significantly higher GPS (p < 0.001), are older, have a lower weight, and decreased BMD in both hip and spine. 50% of participants with MOF are Not-Hispanic White, and 59% without MOF are Black or African-Americans. Rheumatoid arthritis, previous fragility fracture, previous osteoporosis, and parental fracture history significantly differ between MOF and non-MOF participants. In particular, the participants with MOF have higher previous osteoporosis and parental fracture history. 46.9% of participants in the testing dataset had normal BMD (T-score $\ge -1.0$), 37.2% of the participants were osteopenia ($-2.5<$ T-score $< -1.0$), 13.6% of the participants were osteoporosis (T-score $\le -2.5$), and 2.3% met criteria for severe osteoporosis (T-score $\le -2.5$ and presence of one or more fragility fractures).

**Table 1:** Baseline descriptive statistics of 15,776 women in an independent testing dataset stratified by Major osteoporotic fracture (MOF) status

Variable	Participants without MOF (n = 14,835)	Participants with MOF (n = 941)	P-value^*^
Age (years), mean (SD)	61.0 (7.07)	65.5 (7.95)	< 0.001
Height (cm), mean (SD)	162 (5.99)	161 (6.23)	0.095
Weight (kg), mean (SD)	82.0 (17.6)	76.8 (16.4)	< 0.001
Hip BMD (g/cm^2^), mean (SD)	0.927 (0.150)	0.840 (0.129)	< 0.001
Spine BMD (g/cm^2^), mean (SD)	1.03 (0.175)	0.974 (0.167)	< 0.001
GPS^**^, mean (SD)	31.8 (132)	76.0 (147)	< 0.001
GRS^***^, mean (SD)	31.5 (0.420)	31.5 (0.366)	0.009
RACE/ethnicity, $n (\%)$			
American Indian or Alaskan Native	356 (2.5%)	46 (4.9%)	< 0.001
Asian or Pacific Islander	4 (< 1%)	8 (0.9%)	
Black or African-American	8742 (58.9%)	249 (26.5%)	
Hispanic/Latino	2761 (18.6%)	168 (17.9%)	
Not-Hispanic White	2957 (19.9%)	470 (49.8%)	
Smoking, $n (\%)$			
Never Smoked	8672 (58.5%)	472 (50.2%)	< 0.001
Past Smoker	4833 (32.5%)	419 (44.5%)	
Current Smoker	1183 (8.0%)	50 (5.3%)	
Rheumatoid Arthritis, $n (\%)$	1260 (8.5%)	150 (15.9%)	< 0.001
Previous fragility fracture, $n (\%)$	426 (2.9%)	7 (0.7%)	< 0.001
Previous osteoporosis, $n (\%)$	838 (5.6%)	118 (12.5%)	< 0.001
Glucocorticoid use, $n (\%)$	30 (0.2%)	2 (0.2%)	1
Parental fracture history, $n (\%)$	1142 (7.7%)	142 (15.1%)	< 0.001

*SD* standard deviation

^*^P-value was obtained by t-test for continuous variables and chi-square tests for the categorical variable

^**^GPS: Genome-Wide Polygenic Risk Score (LDPred with ρ = 0.03)

^***^GRS: Genetic Risk Score was calculated based on 1103 eBMD-related SNPs

The baseline characteristics of the participants in the independent testing dataset were also stratified by race/ethnicity: Non-Hispanic White (n = 3427), Black or African-American (n = 8991), Hispanic/Latino (n = 2,929), and others (including American Indian or Alaskan Native, and Asian or Pacific Islander) (n = 429). (Additional file 1: Table S1). Among the participants who had at least one MOF in the independent testing dataset (n = 941), the Non-Hispanic White participants had a significantly higher percentage of MOF (49.9%), followed by Black or African-American (26.5%), Hispanic/Latino (17.9%), and others (5.7%). The participants in the Non-Hispanic White group are older and have higher GPS, lower weight, and lower BMD in both hip and spine compared with other racial and ethnic groups.

**Fig. 2:**
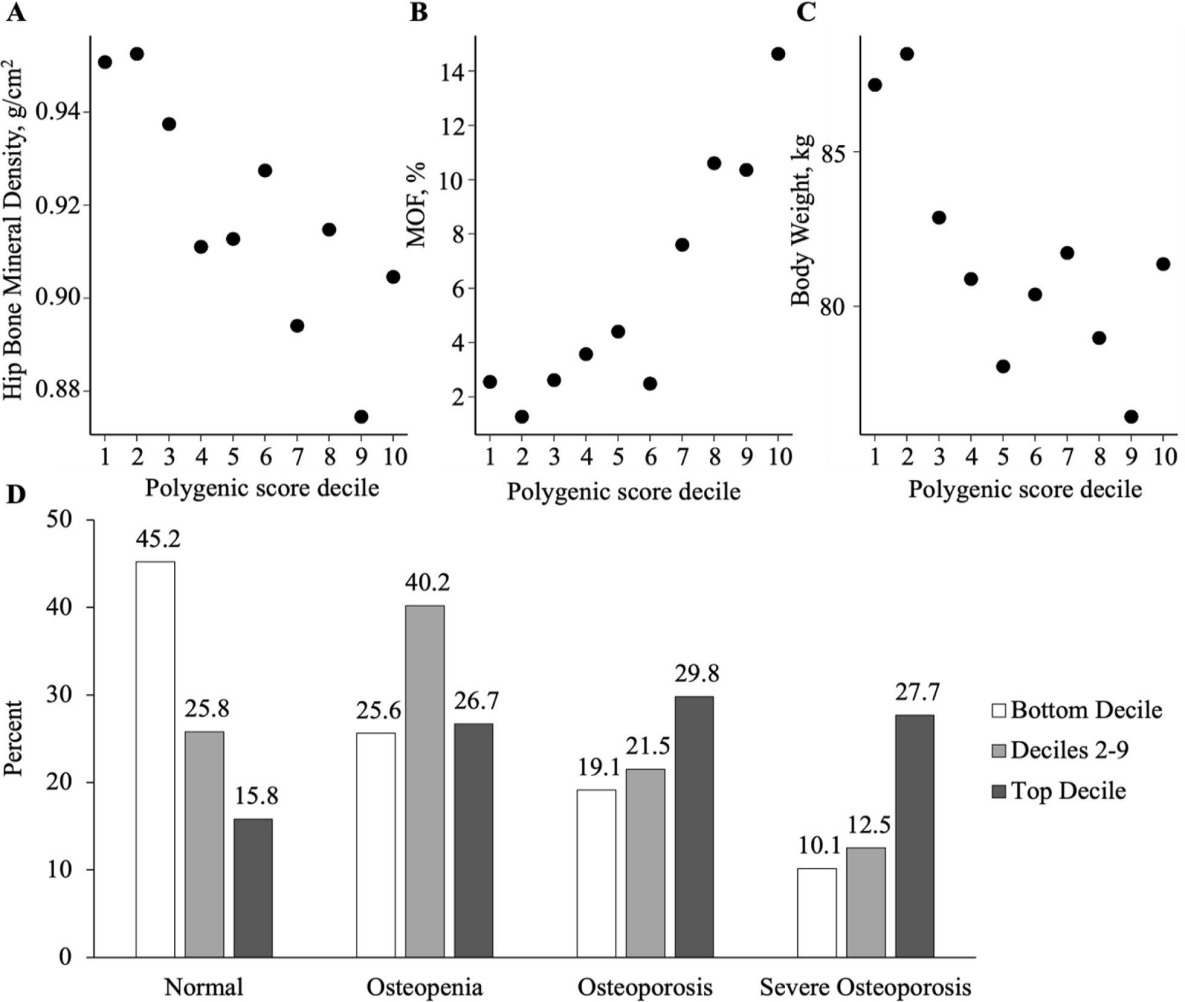
Relationship of a Genome-wide Polygenic Score (GPS, LDPred with $\rho =0.03$) distribution in the testing dataset ($n=\mathrm{15,776})$ with **A** Hip bone mineral density, **B** Major osteoporotic fracture (MOF), and **C** Body weight. Significant differences in osteoporosis categories were observed **D** when participants were stratified into the bottom decile, deciles 2–9, and top decile

**Fig. 3:**
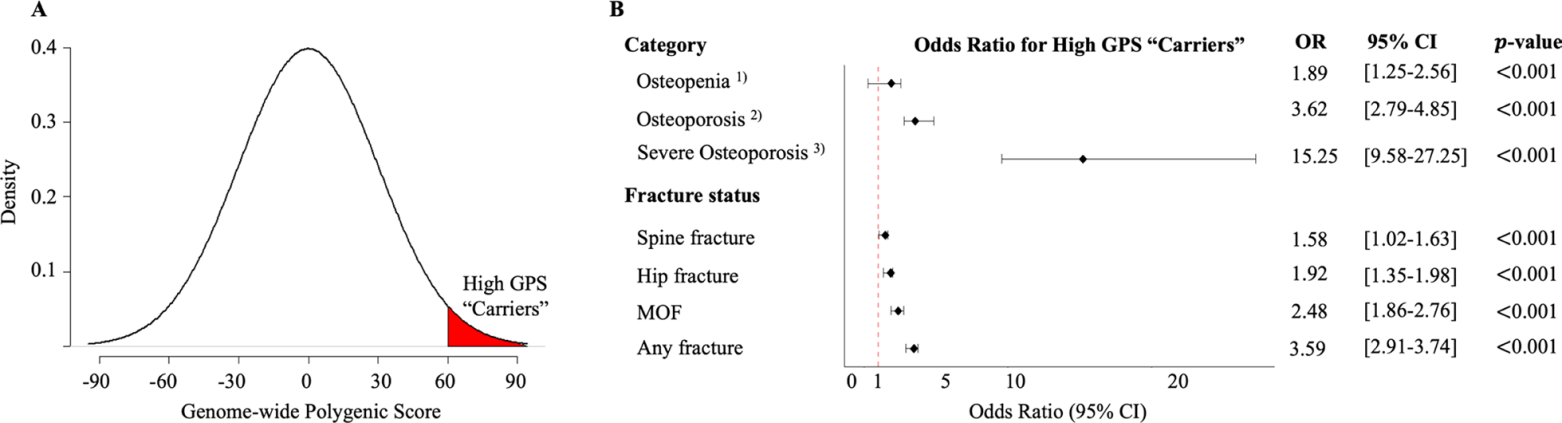
Association of High Genome-wide Polygenic Score (GPS, LDPred with $\rho =0.03$) with osteoporosis category and fracture status in the testing dataset ($n=\mathrm{15,776})$. **A** The top 10% of the GPS distribution was considered high GPS “Carriers,” shaded in red, compared to the remaining 90%. **B** The relationship of high GPS to the osteoporosis category and fracture status was measured using a multiple logistic regression. OR, odds ratio, CI, confidence interval, MOF, major osteoporotic fractures. 1) Osteopenia: T-score between − 1 and − 2.5. 2) Osteoporosis: T-score $\le -2.5$. 3) Severe Osteoporosis: Reserved for patients with a fragility fracture(s) *and* a T-score $\le -2.5$

The GPS approximated a normal distribution in the independent testing dataset (Fig. 3A). The correlation between the GPS and observed BMD (hip and spine) ranged from − 0.225 to − 0.218. These results are similar to our observation in the validation dataset. We then stratified the participants in the testing dataset according to GPS decile and found a remarkable gradient with respect to BMD, MOF, and body weight (Fig. 2A–C). For each decile group, we calculated the mean for the continuous phenotype variable and frequency for the categorical variable. For example, the mean hip BMD was 0.862 g/cm^2^ for those in the top decile of the GPS and 0.931 g/cm^2^ for those in the bottom decile, a difference of 0.069 g/cm^2^ (p < 0.001). Similarly, the average body weight was 76.5 kg for those in the top decile of the GPS and 82.2 kg for those in the bottom decile, a difference of 5.8 kg (p < 0.001). MOF was present in 139 of 941 (14.7%) in the top decile of the GPS versus 27 of 941 (2.8%) in the bottom decile, corresponding to a fivefold gradient in fracture risk (p < 0.001).

Despite the strong associations observed in this study, polygenic susceptibility of the GPS to osteoporosis is not deterministic. Among those in the top decile of the GPS, 57.5% of participants in the testing dataset were osteoporosis and severe osteoporosis (Fig. 2D). In contrast, among those in the bottom decile of the GPS, 29.2 % of participants were osteoporosis and severe osteoporosis. However, among those in the top decile of the GPS, 15.8% had a normal range (Fig. 2D). These results were very similar after adjusting the top ten principal components.

### A high polygenic score is common among those with severe osteoporosis

Conventional analyses of rare genetic mutations are conducted by comparing heterozygous mutation carriers with non-carriers. Individuals carrying the variants within or close to the *LRP5, SOST, OPN,* and *TNFRSF11A* genes had a significantly higher fracture risk, with odds ratios ranging from 1.13 and 1.43 per allele [34]. We tried to mimic this method using the new GPS by labeling the top decile of the GPS distribution as “carriers” and those in the remainder of the distribution as non-carriers (Fig. 3A). The magnitude of risk conferred by a high GPS increased at more extreme levels of observed disease risk. The proportion of high-GPS carriers was 15.8% among the 7410 individuals with normal BMD, 26.7% among the 5872 individuals with osteopenia, 29.8% among the 2140 individuals with osteoporosis, and 27.7% among the 354 individuals with severe osteoporosis. Compared with the remainder of the GPS distribution, the top GPS decile was associated with a 15.25-, 3.62-, and 1.89-fold increased risk of severe osteoporosis, osteoporosis, and osteopenia, respectively (Fig. 3B). Using the same method, we calculated the odds ratio of various fracture types for the top GPS decile versus the 90% remainder of the distribution with adjustment of clinical factors including age, height, weight, parental fracture, previous fracture, smoking, glucocorticoid use, rheumatoid arthritis, BMD (hip or spine), and previous osteoporosis. The results show that compared with the remainder of the GPS distribution, the top GPS decile was associated with a 3.59-, 2.48-, 1.92-, and 1.58-fold increased risk of any-fracture, MOF, hip fracture, and spine fracture, respectively (Fig. 3B).

We further estimated the odds ratio and 95% CI for individuals in the top 30%, 20%, 10%, and 5% of the GPS compared with the remaining individuals (Table 2). The odds ratio per standard deviation increment of MOF risk in the top 5% and 10% of GPS distribution were 3.12 (95% CI 2.25–5.42, p < 0.001) and 2.48 (95% CI 1.86–2.76, p < 0.001), compared with the remaining 95% and 90% of the individuals, respectively. In contrast, the odds ratio per standard deviation increment of MOF risk in the top 5% and 10% of GRS distribution were 1.25 (95% CI 0.98–1.75, p < 0.13) and 0.97 (95% CI 0.67–1.68), respectively.

**Table 2:** The Odds Ratio (OR) estimate of Major osteoporotic fractures (MOF) is derived from two methods in an independent testing dataset ($n=\mathrm{15,776}$)

	GPS (LDPred with ρ = 0.03)		GRS (1,103 SNPs from GWAS)	
	OR (95% CI)	$p$-value	OR (95% CI)	$p$-value
Top 30% vs. Remaining 70%	1.67 (0.94, 2.82)	0.06	0.72 (0.32, 1.42)	0.38
Top 20% vs. Remaining 80%	2.36 (1.42, 3.80)	0.01	0.82 (0.38, 1.56)	0.57
Top 10% vs. Remaining 90%	2.48 (1.86, 2.76)	< 0.001	0.97 (0.67, 1.68)	0.11
Top 5% vs. Remaining 95%	3.12 (2.25, 5.42)	< 0.001	1.25 (0.98, 1.75)	0.13

The odds ratio (OR) was calculated for the top 30%, 20%, 10%, and 5% of the GPS and GRS compared with the remaining individuals. *CI* confidence interval. The odds ratios were calculated in a multiple logistic regression model adjusted for the clinical risk factors of age, height, weight, parental fracture, previous fracture, smoking, glucocorticoid use, rheumatoid arthritis, hip BMD, and previous osteoporosis. In a separate analysis, we replaced spine BMD with hip BMD. The results were similar, not shown in this table

We also examined the transferability of GPS by comparing the odds ratio and its 95% CI stratified by four populations: Non-Hispanic White (n = 3427), Black or African-American (n = 8991), Hispanic/Latino (n = 2929) and others (including American Indian or Alaskan Native, and Asian or Pacific Islander) (n = 429). (Additional file 1: Table S2). The odds ratio per standard deviation increment of MOF risk in the top 5% of GPS distribution in the Non-Hispanic White, Black or African American, and Hispanic/Latino were 2.26 (95% CI 1.56–2.63, p < 0.001), 1.53 (95% CI 1.13–1.84, p < 0.001) and 1.19 (95% CI 1.01–1.47, p < 0.001), compared to the remaining 95% of GPS distribution, respectively.

### Postmenopausal women’s risk of developing severe osteoporosis varies according to polygenic score

**Fig. 4:**
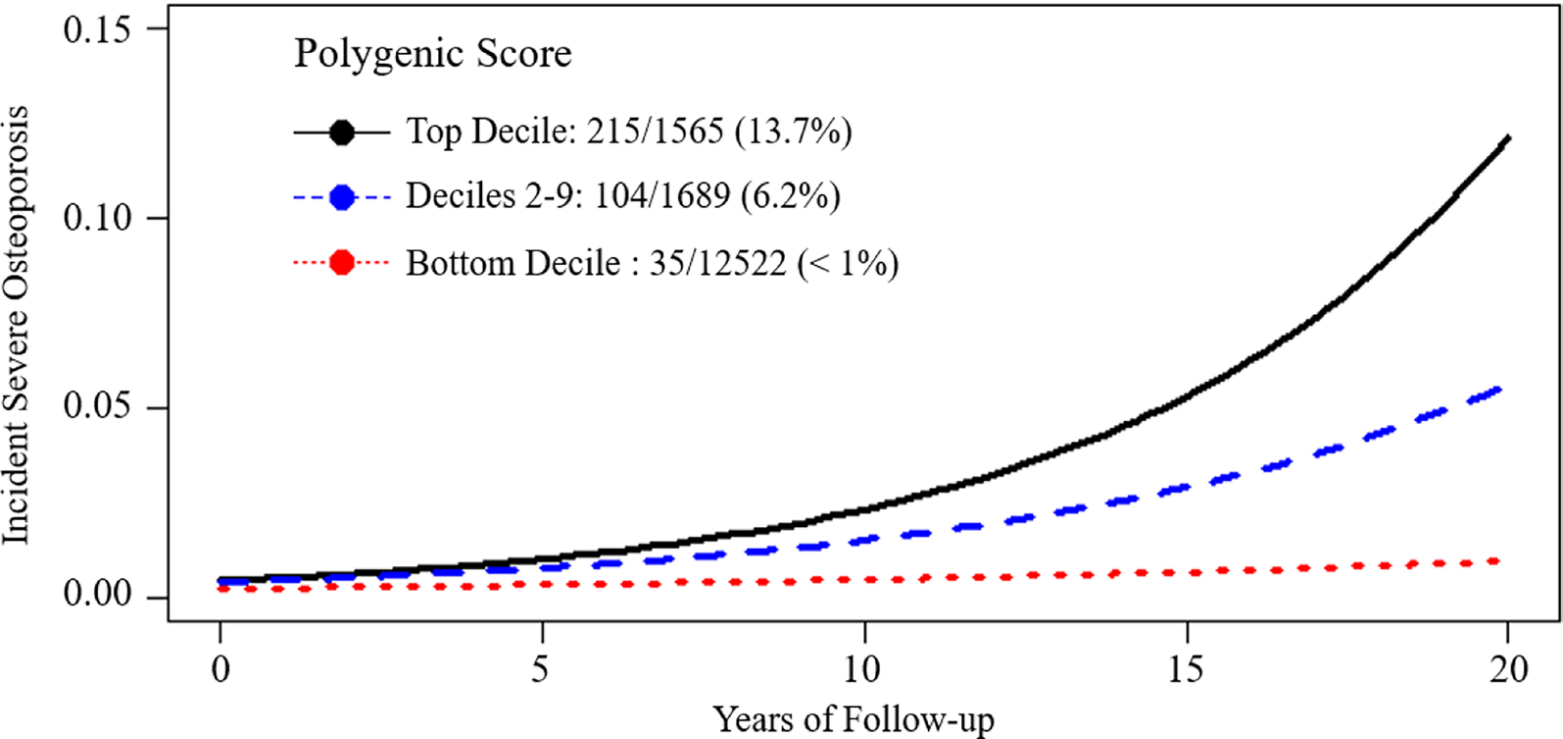
Association of High Genome-wide Polygenic Score (GPS, LDPred with $\rho =0.03$) with incident severe osteoporosis. Among 15,776 individuals in an independent testing dataset, GPS was stratified into top decile, deciles 2–9, and bottom decile. Incident severe osteoporosis is plotted with three GPS group stratification

Although only a small percentage of postmenopausal women experienced severe osteoporosis in their middle age (around 50) at the baseline, the prevalence of severe osteoporosis increases substantially over subsequent decades at the 20-year follow-up. We hypothesized that the GPS might significantly predict who will develop severe osteoporosis during the transition from middle age to the elderly. Among individuals in the top decile of the GPS, 215 of 1565 (13.7%) developed severe osteoporosis compared with 6.2% of those in deciles 2–9 (Fig. 4). By contrast, among those in the lowest decile, only 35 of 12,522 (< 1%) individuals developed severe osteoporosis.

**Fig. 5:**
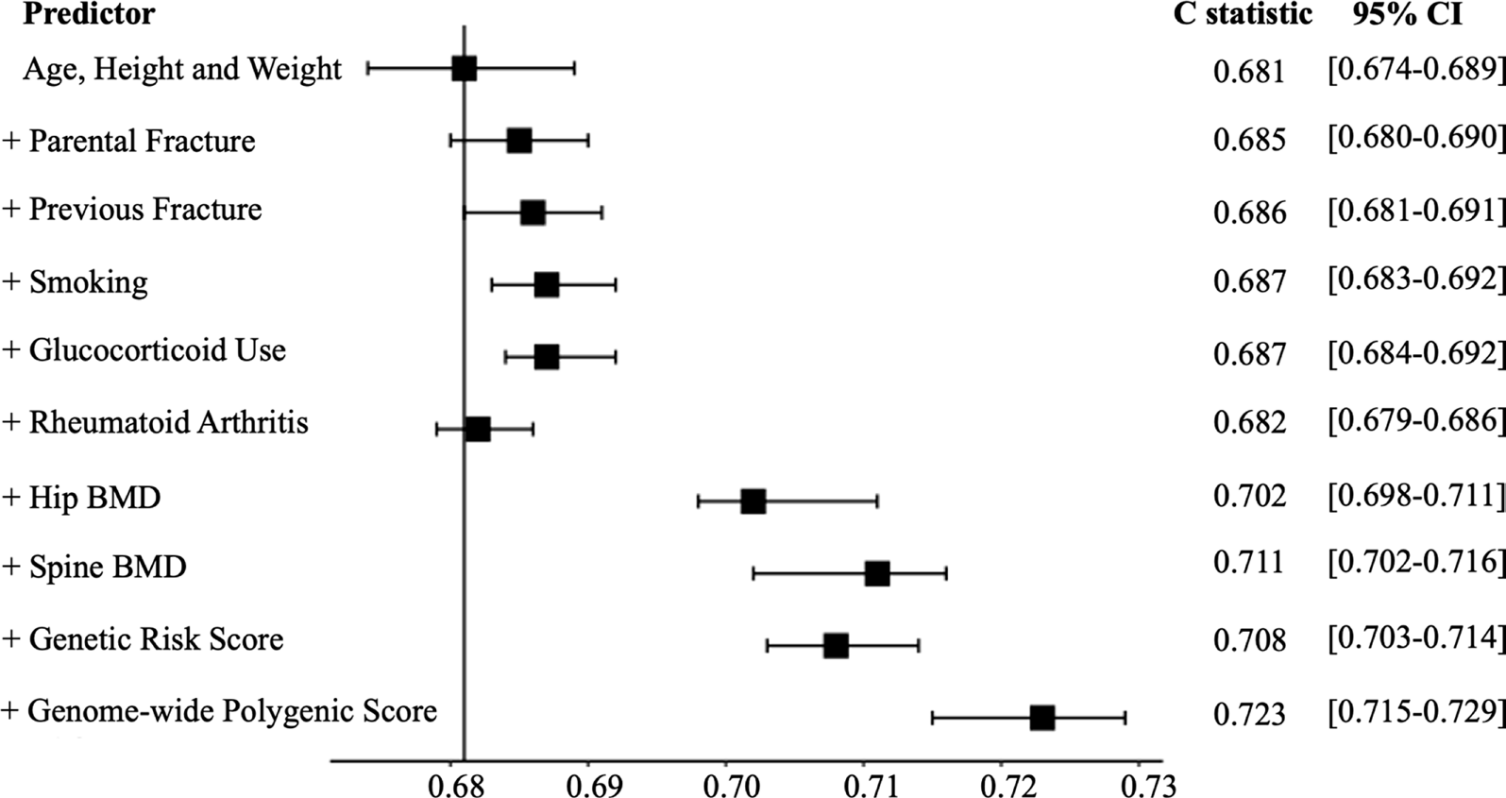
Discriminative capacity of the genome-wide polygenic score and clinical risk factors in the testing dataset ($n=\mathrm{15,776})$. The C statistic estimate of major osteoporotic fractures is first obtained with a baseline model of age, height, and weight using a multiple logistic regression model. Next, the C statistic was calculated after the additional inclusion of individual clinical risk factors; parental fracture, previous fracture, smoking, glucocorticoid use, rheumatoid arthritis, hip bone mineral density, spine bone mineral density, genetic risk score (1103 SNPs from GWAS), and genome-wide polygenic score (LDPred with ρ = 0.03)

With respect to the discriminative capacity, we first evaluated a baseline model of age, height, and weight, yielding a C statistic of 0.681 (95% CI 0.674–0.689). Each of the nine additional risk factors was then added (individually) to this baseline model for the outcome of major osteoporotic fractures (Fig. 5). GPS had a higher discriminative capacity with a C statistic of 0.723 (95% CI 0.715–0.729). By contrast, the addition of GRS has a C statistic of 0.708 (95% CI 0.703–0.714).

## Discussion

We demonstrate a systematic approach to deriving and validating a GPS, incorporating information from 103,155 common genetic variants, to predict polygenic susceptibility to osteoporosis and osteoporotic fracture. We tested the GPS in 15,776 participants from an independent testing dataset, including three cohorts. The GPS we derived herein remarkably improved the prediction of BMD, severe osteoporosis, and major osteoporotic fractures in middle-aged postmenopausal women. The extreme of the GPS distribution inheriting susceptibility to osteoporotic fracture risk is equivalent to an individual who carries the variants within or close to the *LRP5, SOST, OPN*, and *TNFRSF11A* genes. Moreover, the GPS was strongly associated with a gradient in BMD that started to emerge in five years of follow-up and showed more enormous differences in fracture risk in subsequent years of follow-up (Fig. 4). Our finding suggests that although the relative risk of severe osteoporosis for postmenopausal women is relatively similar at age around 50 (baseline), the cumulative incident is significantly different between high (the top decile of the GPS) and lower-risk groups (the bottom decile of the GPS) at the 20 years of follow-up.

The GPS far and significantly outperformed a conventional GRS based only on the 1103 conditionally independent genetic variants that have a genome-wide significant association with fracture and estimated BMD. The findings with the novel GPS are more consistent with the highly polygenic nature of BMD and osteoporosis. For example, in a direct comparison in an independent testing dataset of 15,776 participants, the correlation of the GPS with observed hip BMD was − 0.225 compared with − 0.146 for the 1103 genetic variants score. Similarly, the correlation of the GPS with observed spine BMD was − 0.218 compared with − 0.159 with the GRS derived from the 1103 SNPs.

The performance of the new GPS using a genome-wide set of 103,155 variants was substantially improved, which was anticipated by a previous theoretical projection study that analyzed early GWAS findings [35]. Our results suggested minimal “missing heritability” of BMD when accounting for the entire range of discovered genetic variation. In the present study, we employed a newly developed computational algorithm that can explicitly model the correlation structure between genetic variants in calculating the weight of each variant [11]. This new algorithm has been demonstrated to outperform a number of prior methods for a range of complex diseases and traits, including colorectal cancer [36] and Alzheimer’s disease [37].

The new GPS has several advantages over a conventional GRS. First, the novel GPS includes more associated SNPs in the linkage disequilibrium region where a significant SNP lead is located. Second, several studies showed that genetic correlation matters [38–40]; unlike conventional GRS, our new GPS has accounted for correlations between genetic variants. Third, osteoporosis is a polygenic disorder where several genes contribute with relatively modest effects on bone mass, microstructure, and other determinants of fracture risk [41]. It is well-known that many complex diseases contain multiple-associated loci on the same chromosome [42]. Our results are consistent with a liability threshold model where the probability of any given pathogenic variant carrier crossing the threshold into the disease is influenced by the underlying liability conferred by the polygenic background [43]. Thus, accounting for polygenic susceptibility is likely to increase the accuracy of osteoporotic fracture risk estimation.

Recently, Lu et al. [44] found that a polygenetic score derived from heel quantitative speed of sound performed better than other clinical risk factors and improved fracture risk prediction. Similarly, studies on other disease outcomes found that GPS performs better than conventional GRS in assessing the risk of colorectal cancer ^12^ and Alzheimer’s disease ^13^. Our results in comparing GPS with GRS in fracture risk prediction are consistent with these observations. The limitations of these conventional GRSs could be due to multiple reasons. The conventional GRS included SNPs restricted to the genome-wide significant levels and did not consider the genetic correlation between genetic variants [38]. Notably, the osteoporosis-related GRS may not sufficiently capture the underlying genetic predisposition of osteoporotic fractures where several genes contribute with relatively modest effects from multiple genome locations [41]. Thus, these conventional GRSs only have the capacity to account for a small proportion of variance in disease risk [45]. Our study demonstrated that the novel polygenetic risk score developed herein significantly improves osteoporotic fracture prediction and risk assessment.

To take advantage of participant diversity in our independent testing dataset, we examined the transferability of GPS in several racial/ethnic groups. We found that the new GPS has a better fracture prediction in the Non-Hispanic White group than Black/African American and Hispanic/Latino. The underlying reasons are that the new GPS was derived from GWAS summary statistics of the Non-Hispanic White study sample, and a European reference panel was used in the GPS development. A few other studies have reported GRS transferability of other disease risks in different populations; however, these studies only examined the GRS based on a limited number of variants, e.g., tens to a few hundred genetic variants [46, 47]. To the best of our knowledge, the present study is the first attempt to examine the transferability of osteoporosis-related GPS in various racial and ethnic groups. We found that GPS derived from the study sample, consisting mainly of Caucasians, had a lower prediction power for fracture risk in minority populations. Our observations are consistent with a recent study [48] focused on type 2 diabetes and coronary artery disease.

Our study should be interpreted in light of a few limitations. First, although our GPS strongly associates osteoporosis and fracture, it was developed from the non-Hispanic White population; additional genetic discovery studies, including sufficient other racial/ethnic study samples, are warranted to derive GPS that is more generalizable to minorities. However, comprehensive GWAS studies that included sufficient minority participants are still lacking. Secondly, because the study population of WHI only includes postmenopausal women, caution should be taken when applying our findings to pre-menopausal women and men. Lastly, we should alert readers that other sample-related factors such as age, sample ascertainment, or variation in other clinical risk factors may affect the transferability of our GPS in different patient groups [49].

In the era of precision medicine, one of our goals is to accurately predict disease risk based on an individual’s genetic information [50]. With advanced genomic sequencing technology, more genetic variants will be discovered, and genetic information for more individuals, including racial/ethnical minorities, will be more accessible. We expect that the newly developed GPS can be utilized in healthcare to identify individuals who inherit high susceptibility before the related clinical diseases manifest. These patients may otherwise not be identified early on using existing tools. Early detection can help mitigate the disease burden with low-cost lifestyle changes or more frequent screening [51]. The novel GPS could also be utilized to identify low-risk individuals who might otherwise be enrolled unnecessarily in more frequent conventional screening based on age and other clinical risk factors. However, further genetic discovery research in minority groups is warranted to improve the transferability of the updated GPS in diverse patients. Our GPS can be updated with more genetic discoveries in diverse populations and further optimize trans-ancestry polygenic fracture prediction.

## Conclusions

In summary, we developed and validated a comprehensive GPS with a substantially higher capacity for predicting osteoporotic fracture risk. Moreover, the new GPS can potentially improve identifying both high and low-risk patients in clinical settings. More GWASs that focus on minority populations are warranted for future studies to improve the transferability of this new GPS across diverse populations.

## Supplementary Information

**Additional file 1:** **Table S1. **Baseline descriptive statistics of 15,776 women in an independent testing dataset stratified by Non-Hispanic White, Black or African-American, Hispanic/Latino, and Others (including American Indian or Alaskan Native, and Asian or Pacific Islander). **Table S2**. The Odds Ratio (OR) estimate of Major osteoporotic fractures (MOF) is derived from the GPS (LDPred with ρ=0.03), stratified by four populations: Non-Hispanic White (n = 3,427), Black or African-American (n=8,991), Hispanic/Latino (n=2,929) and others (including American Indian or Alaskan Native, and Asian or Pacific Islander) (n = 429). The odds ratio (OR) was calculated for the top 30%, 20%, 10%, and 5% of the GPS and GRS compared with the remaining individuals. CI, confidence interval. **Figure S1**. Correlations between hip bone mineral density (BMD) and genome-wide polygenic score (GPS) with a different fraction of causal variants ($\rho )$; A ($\rho =0.001)$, B ($\rho =0.003)$, C ($\rho =0.01)$, D ($\rho =0.03)$, E ($\rho =0.1)$, F ($\rho =0.3)$, and G ($\rho =1)$ in a validation dataset of 2,458 participants from the GARNET WHI sub-study. **Figure S2.** Correlations between spine bone mineral density (BMD) and genome-wide polygenic score (GPS) with a different fraction of causal variants ($\rho )$; A ($\rho =0.001)$, B ($\rho =0.003)$, C ($\rho =0.01)$, D ($\rho =0.03)$, E ($\rho =0.1)$, F ($\rho =0.3)$, and G ($\rho =1)$ in a validation dataset of 2,458 participants from the GARNET WHI sub-study.

## Data Availability

The data used in the current study is publically available through the database of Genotype and Phenotype (dbGap) (https://www.ncbi.nlm.nih.gov/projects/gap/cgi-bin/study.cgi?study_id=phs000200.v12.p3). The summary statistics used in the current study are available from the GEFOS Consortium at http://www.gefos.org/?q=content/data-release-2018

## Abbreviations

BMD: Bone mineral density
CRF: Clinical risk factors
dbGap: The database of genotypes and phenotypes
GEFOS: GEnetic Factors for OSteoporosis Consortium
GRS: Genetic risk score
GPS: Genome-wide polygenic score
GWAS: Genome-wide association study
LD: Linkage disequilibrium
LR: Logistic regression
MOF: Major osteoporotic fractures
SNPs: Single Nucleotide Polymorphisms
WHI: Women’s Health Initiative
WHI OS: Women’s Health Initiative Observational Study
WHI CT: Women’s Health Initiative Clinical Trials
