# Supplementary material for: Genome-wide polygenic risk score for major osteoporotic fractures in postmenopausal women using associated single nucleotide polymorphisms
Source: J Transl Med. 2023 Feb 16;21:127. doi: 10.1186/s12967-023-03974-2 (PMC9933300; doi:10.1186/s12967-023-03974-2)
Supplement: Supplementary file 1 — Additional file 1: Table S1. Baseline descriptive statistics of 15,776 women in an independent testing dataset stratified by Non-Hispanic White, Black or African-American, Hispanic/Latino, and Others (including American Indian or Alaskan Native, and Asian or Pacific Islander). Table S2. The Odds Ratio (OR) estimate of Major osteoporotic fractures (MOF) is derived from the GPS (LDPred with ρ=0.03), stratified by four populations: Non-Hispanic White (n = 3,427), Black or African-American (n=8,991), Hispanic/Latino (n=2,929) and others (including American Indian or Alaskan Native, and Asian or Pacific Islander) (n = 429). The odds ratio (OR) was calculated for the top 30%, 20%, 10%, and 5% of the GPS and GRS compared with the remaining individuals. CI, confidence interval. Figure S1. Correlations between hip bone mineral density (BMD) and genome-wide polygenic score (GPS) with a different fraction of causal variants ($\rho )$ρ); A ($\rho =0.001)$ρ=0.001), B ($\rho =0.003)$ρ=0.003), C ($\rho =0.01)$ρ=0.01), D (\d [file 12967_2023_3974_MOESM1_ESM.docx]

# Additional file Table

**Table S1.** Baseline descriptive statistics of 15,776 women in an independent testing dataset stratified by Non-Hispanic White, Black or African-American, Hispanic/Latino, and Others (including American Indian or Alaskan Native, and Asian or Pacific Islander).

| **Variable** | **Participants without MOF  (n=14,835)** | | | |  | **Participants with MOF  (n=941)** | | | |
| --- | --- | --- | --- | --- | --- | --- | --- | --- | --- |
|  | **NHW (n=2,957)** | **BAA (n=8,742)** | **HL**  **(n=2,761)** | **Others* (n=375)** |  | **NHW (n=470)** | **BAA (n=249)** | **HL**  **(n=168)** | **Others* (n=54)** |
| **Age (years),** mean (SD) | 65.0 (6.90) | 60.1 (6.73) | 60.0 (6.82) | 59.1 (6.53) |  | 69.0 (6.73) | 63.3 (7.10) | 62.4 (7.67) | 55.4 (5.89) |
| **Height (cm)**, mean (SD) | 161 (6.04) | 163 (5.78) | 159 (5.43) | 162 (5.99) |  | 161 (6.29) | 163 (4.64) | 159 (5.29) | 161 (10.6) |
| **Weight (kg)**, mean (SD) | 77.9 (16.7) | 86.1 (17.7) | 73.5 (13.9) | 80.4 (18.7) |  | 72.2 (11.7) | 86.5 (19.2) | 76.0 (16.4) | 74.5 (18.2) |
| **Hip BMD (g/cm^2^)**, mean (SD) | 0.86 (0.14) | 0.97 (0.14) | 0.87 (0.14) | 0.89 (0.14) |  | 0.78 (0.11) | 0.95 (0.12) | 0.84 (0.10) | 0.84 (0.08) |
| **Spine BMD (g/cm^2^)**, mean (SD) | 0.97 (0.16) | 1.08 (0.18) | 0.98 (0.15) | 0.97 (0.16) |  | 0.92 (0.15) | 1.10 (0.11) | 0.97 (0.21) | 0.88 (0.06) |
| **GPS****, mean (SD) | 180 (28.7) | 1.95 (109) | 33.2 (62.9) | 54.7 (78.7) |  | 220 (38.2) | 8.90 (110) | 41.2 (106) | 73.8 (28.7) |
| **GRS*****, mean (SD) | 31.6 (0.45) | 31.5 (0.41) | 31.6 (0.41) | 31.7 (0.37) |  | 31.5 (0.41) | 31.4 (0.23) | 31.6 (0.38) | 31.7 (0.33) |
| **Smoking,** $\boldsymbol{n (\%)}$  Never Smoked | 1840 (62.2%) | 4812 (55.0%) | 1797 (65.1%) | 223 (59.5%) |  | 222 (47.2%) | 75 (30.1%) | 135 (80.4%) | 40 (74.1%) |
| Past Smoker | 969 (32.8%) | 2981 (34.1%) | 758 (27.5%) | 125 (33.3%) |  | 240 (51.1%) | 139 (55.8%) | 26 (15.5%) | 14 (25.9%) |
| Current Smoker | 142 (4.8%) | 847 (9.7%) | 172 (6.2%) | 22 (5.9%) |  | 8 (1.7%) | 35 (14.1%) | 7 (4.2%) | 0 (0%) |
| **Rheumatoid Arthritis,** $\boldsymbol{n (\%)}$ | 97 (3.3%) | 874 (10.0%) | 237 (8.6%) | 52 (13.9%) |  | 86 (18.3%) | 51 (20.5%) | 6 (3.6%) | 7 (13.0%) |
| **Previous fragility fracture,** $\boldsymbol{n (\%)}$ | 47 (1.6%) | 241 (2.8%) | 112 (4.1%) | 26 (6.9%) |  | 0 (0%) | 0 (0%) | 7 (4.2%) | 0 (0%) |
| **Previous osteoporosis,** $\boldsymbol{n (\%)}$ | 148 (5.0%) | 417 (4.8%) | 255 (9.2%) | 18 (4.8%) |  | 44 (9.4%) | 59 (23.7%) | 15 (8.9%) | 0 (0%) |
| **Glucocorticoid use,** $\boldsymbol{n (\%)}$ | 3 (0.1%) | 23 (0.3%) | 4 (0.1%) | 0 (0%) |  | 1 (0.2%) | 1 (0.4%) | 0 (0%) | 0 (0%) |
| **Parental fracture history,** $\boldsymbol{n (\%)}$ | 344 (11.6%) | 439 (5.0%) | 332 (12.0%) | 27 (7.2%) |  | 121 (25.7%) | 0 (0%) | 19 (11.3%) | 2 (3.7%) |

* Others includes American Indian or Alaskan Native, and Asian or Pacific Islander.

** GPS: Genome-Wide Polygenic Risk Score.

*** GRS: Genetic Risk Score, which was calculated based on 1,103 eBMD-related SNPs.

NHW: Non-Hispanic White, BAA: Black or African-American, HL: Hispanic/Latino, SD: standard deviation

**Table S2**. The Odds Ratio (OR) estimate of Major osteoporotic fractures (MOF) is derived from the GPS (LDPred with ρ=0.03), stratified by four populations: Non-Hispanic White (n = 3,427), Black or African-American (n=8,991), Hispanic/Latino (n=2,929) and others (including American Indian or Alaskan Native, and Asian or Pacific Islander) (n = 429). The odds ratio (OR) was calculated for the top 30%, 20%, 10%, and 5% of the GPS and GRS compared with the remaining individuals. CI, confidence interval.

|  | **Non-Hispanic White**  **(n = 3,427)** | |  | **Black or African American**  **(n = 8,991)** | |  | **Hispanic/Latino**  **(n=2,929)** | |  | **Others***  **(n = 429)** | |
| --- | --- | --- | --- | --- | --- | --- | --- | --- | --- | --- | --- |
|  | OR  (95% CI) | $p$-value |  | OR  (95% CI) | $p$-value |  | OR  (95% CI) | $p$-value |  | OR  (95% CI) | $p$-value |
| Top 30% vs. Remaining 70% | 1.32  (0.75, 1.96) | 0.15 |  | 1.04  (0.69, 1.24) | 0.16 |  | 0.95  (0.58, 1.19) | 0.22 |  | 0.87  (0.51, 1.09) | 0.29 |
| Top 20% vs. Remaining 80% | 1.56  (0.96, 2.03) | 0.12 |  | 1.12  (0.76, 1.41) | 0.29 |  | 1.01  (0.71, 1.36) | 0.18 |  | 0.93  (0.68, 1.23) | 0.25 |
| Top 10% vs. Remaining 90% | 1.97  (1.12, 2.38) | <0.001 |  | 1.26  (0.91, 1.52) | 0.13 |  | 1.14  (0.86, 1.47) | 0.14 |  | 1.02  (0.91, 1.22) | 0.18 |
| Top 5% vs. Remaining 95% | 2.26  (1.56, 2.63) | <0.001 |  | 1.53  (1.13, 1.84) | <0.001 |  | 1.19  (1.01, 1.47) | <0.001 |  | 1.09  (0.99, 1.48) | 0.09 |

The odds ratios were calculated in a multiple logistic regression model adjusted for the clinical risk factors of age, height, weight, parental fracture, previous fracture, smoking, glucocorticoid use, rheumatoid arthritis, hip BMD, and previous osteoporosis. In a separate analysis, we replaced spine BMD with hip BMD. The results were similar, not shown in this table.

* Others include American Indian or Alaskan Native and Asian or Pacific Islander.

# Additional file Figure

**Figure S1**. Correlations between hip bone mineral density (BMD) and genome-wide polygenic score (GPS) with a different fraction of causal variants ($\rho)$; A ($\rho=0.001)$, B ($\rho=0.003)$, C ($\rho=0.01)$, D ($\rho=0.03)$, E ($\rho=0.1)$, F ($\rho=0.3)$, and G ($\rho=1)$ in a validation dataset of 2,458 participants from the GARNET WHI sub-study.

| **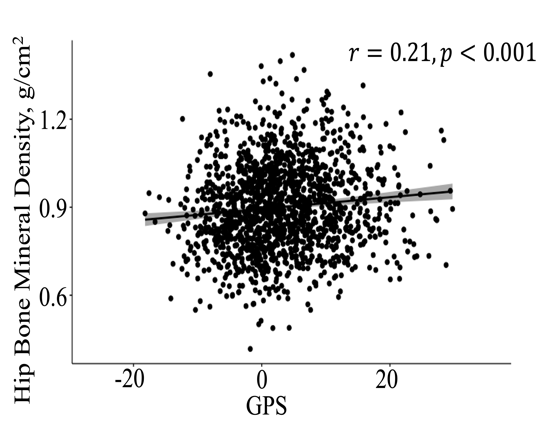A** ($\rho=0.001)$ | **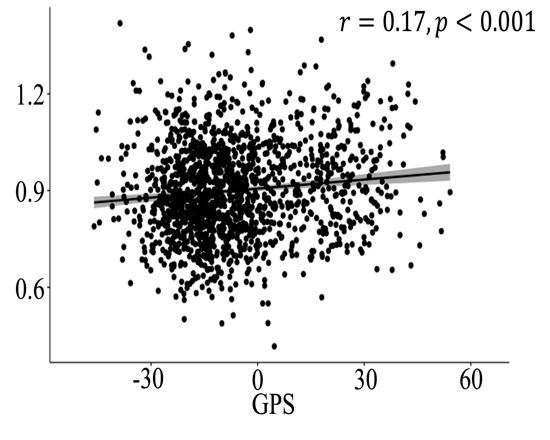B** ($\rho=0.003)$ | **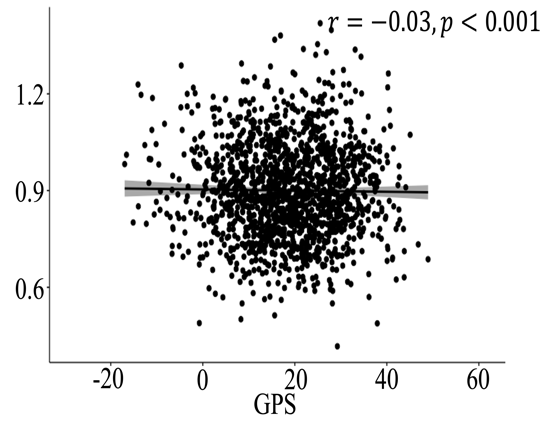C** ($\rho=0.01)$ | **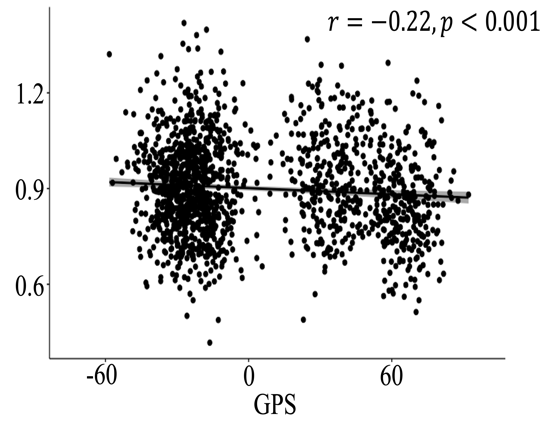D** ($\rho=0.03)$ |
| --- | --- | --- | --- |
| **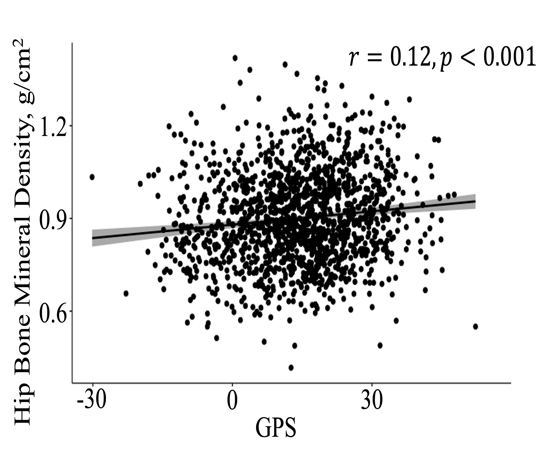E** ($\rho=0.1)$ | **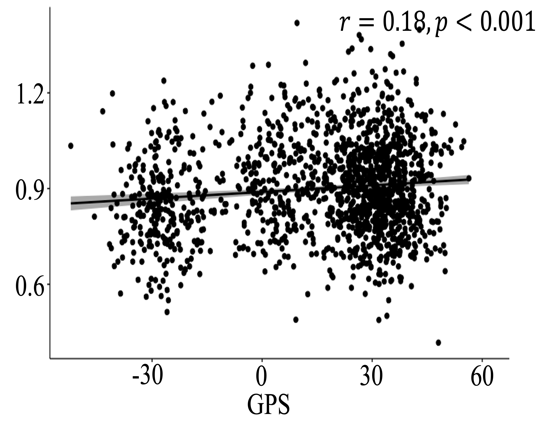F** ($\rho=0.3)$ | **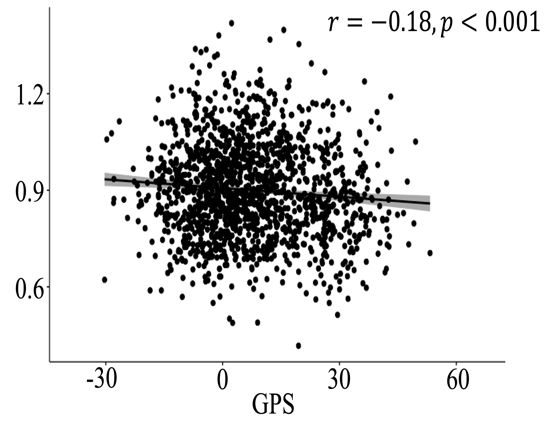G** ($\rho=1)$ |  |

**Figure S2**. Correlations between spine bone mineral density (BMD) and genome-wide polygenic score (GPS) with a different fraction of causal variants ($\rho)$; A ($\rho=0.001)$, B ($\rho=0.003)$, C ($\rho=0.01)$, D ($\rho=0.03)$, E ($\rho=0.1)$, F ($\rho=0.3)$, and G ($\rho=1)$ in a validation dataset of 2,458 participants from the GARNET WHI sub-study.

| **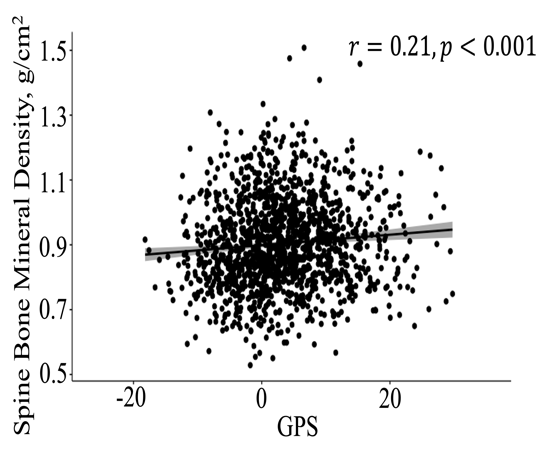A** ($\rho=0.001)$ | **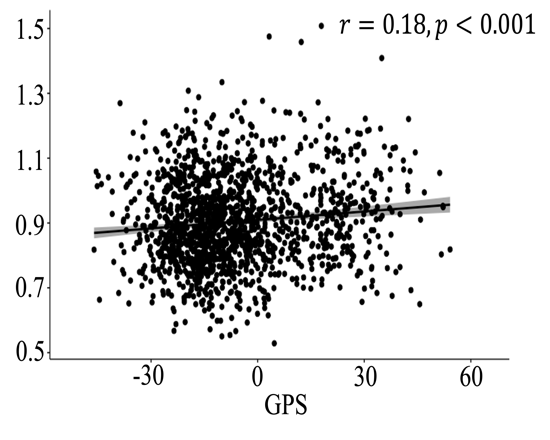B** ($\rho=0.003)$ | **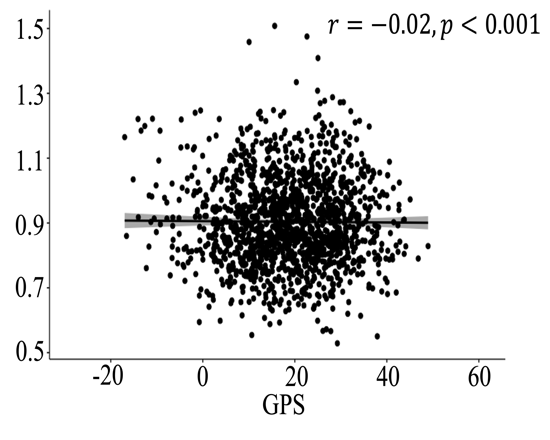C** ($\rho=0.01)$ | **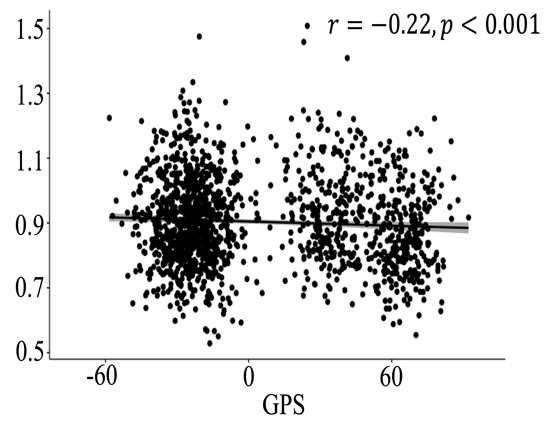D** ($\rho=0.03)$ |
| --- | --- | --- | --- |
| **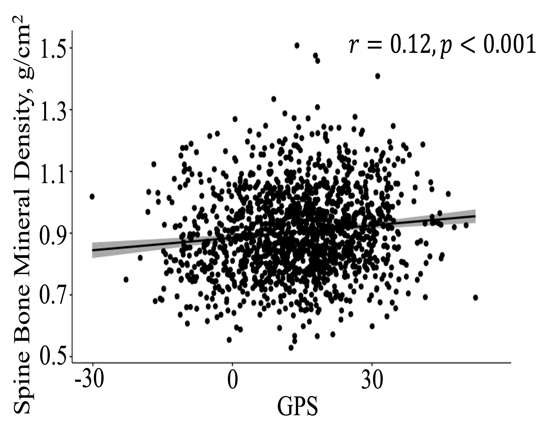E** ($\rho=0.1)$ | **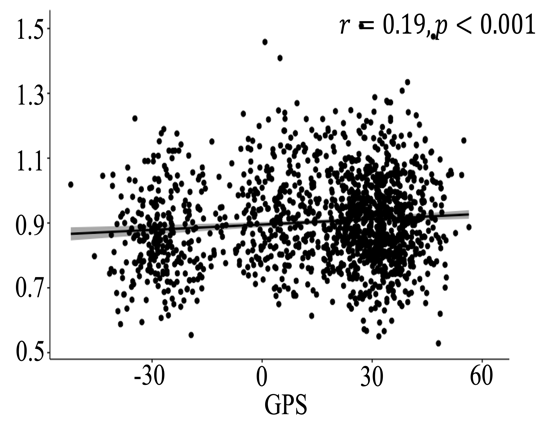F** ($\rho=0.3)$ | **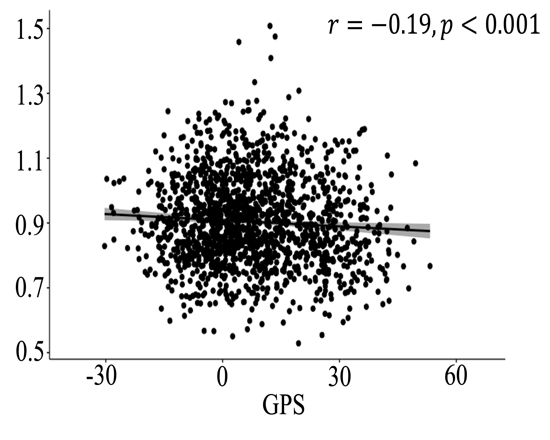G** ($\rho=1)$ |  |
